# The influence of ergonomic breastfeeding training on some health parameters in infants and mothers: a randomized controlled trial

**DOI:** 10.1186/s13690-019-0373-x

**Published:** 2019-11-11

**Authors:** Raha Afshariani, Marjan Kiani, Zahra Zamanian

**Affiliations:** 1 International Board Certified Lactation Consultant (IBCLC), Advanced Lactation Consultant (ALC), Faculty of Shiraz University of Medical Sciences, Shiraz, Iran; 2 Department of Ergonomics, School of Health, Shiraz University of Medical Sciences, Shiraz, Iran; 3 Department of Ergonomics, School of Health, Shiraz University of Medical Sciences, Shiraz, 1433671348 Iran

**Keywords:** Breastfeeding, Ergonomics, Training, Musculoskeletal disorders

## Abstract

**Background:**

Breastfeeding is considered as a protective factor against non-communicable diseases in infants and mothers. The first aim of this study was to assess the influence of ergonomic breastfeeding training on the infants’ growth. The second aim was to investigate the effectiveness of this training for improving musculoskeletal disorders in mothers.

**Methods:**

In this randomized control trial, 104 participants who were referred to the health centers in Shiraz for breastfeeding care were randomly allocated into the intervention or comparison group using a size-four block sampling method. At birth, 2, 4, and 6 months later childbirth, Rapid Upper Limb Assessment was used to assess musculoskeletal disorders in participants**,** and the severity of their discomfort was measured with the Visual Analog Scale. During the 6 months of the study (March to September, 2017), the weight and height of the neonates were measured every 2 months.

**Results:**

Significant differences were found between groups in the priority level for corrective action in mothers’ postures determined by Rapid Upper Limb Assessment at 2, 4 and 6 months after childbirth (*p* < 0.001). Based on the Visual Analog Scale results in mothers at 6 months after childbirth, fewer back pain was reported by the intervention group (*p* = 0.03). No significant difference were found in the infants^’^ weights and heights in boys and girls at all growing stages between the two study groups (*p* > 0.05). However, the mean height for age of the girl infants at age of 6 months was higher among intervention groups compared to the controls (*p* = 0.01).

**Conclusion:**

This study demonstrated that ergonomic breastfeeding training reduced the incidence of musculoskeletal disorders in mothers but seems not to have any significant and consistent impact on the infants’ growth.

**Trial registration:**

fa.irct.ir IRCT2014042317398N1.

## Background

Exclusive breastfeeding (EBF) is defined as feeding an infant with only human milk for the first 6 months of life without routine replacement of any meal by supplemental feeding [1, 2]. Fewer than half of newborns globally are exclusively breastfed for the first 6 months of life [3, 4].

Breastfeeding has many advantages for both infants and mothers [5, 6]. The nutritional value of human milk is important for strengthening the infant’s immune system and protecting them against various infections, including diarrheic diseases and respiratory infections [7, 8]. Breastfeeding also reduces medical costs, formula expenses and hospitalization [7, 8]. Despite the great benefits of human milk for the infant’s health, breastfeeding rates are low throughout worldwide, as well as in Iran [9]. The rate of EBF up to 6 months of age in Angola was 11% and in America, Egypt, Pakistan, Saudi Arabia, Iraq, and Iran it was 28, 53, 37, 31, 25 and 21%, respectively [3]. Indeed, a meta-analysis by Ranjbaran et al. reviewed 16 studies and estimated the overall prevalence of exclusive breastfeeding in the first 6 months post birth to be 49.1% [7]. Thus, identifying factors that can increase the rate of breastfeeding has been of great interest to national and international health and social authorities [10, 11].

The mother’s body posture during breastfeeding is very important. Different methods are suggested for breastfeeding mothers to adopt, including the cross-cradle, under-arm, side-lying, and supine (lying face upward) positions. The under-arm posture (or football hold) is an appropriate position for feeding premature babies. It enables mothers to watch the infant’s face better and have more control of the infant’s head. In the cross-cradle position, using a pillow can be helpful to support the infant and raise it to the same level as the breast. In the laying down method, the infant and mother both lie down, tummy-to-tummy. In this method, the mother puts her arm above the baby’s head and bends it under her own head. Her other hand wraps around the infant to pull it close. This is the best posture for close feeding when the mother is lying down [12]. Among the different breastfeeding methods, the cross cradle position is the most common one, in which the baby is held close to the mother: the ear, shoulder, and hip of the baby are aligned, the head and shoulder of the baby are supported, and the infant’s nose faces the breast opposite the nipple [13].

Numerous studies have been conducted to identify factors that affect mothers during breastfeeding, for instance, infant’s gender and age, mother’s level of education, maternal occupation and initial time of breastfeeding postpartum [14–17]. Socioeconomic and cultural status and immediate mother–infant skin-to-skin contact (SSC) seem to be the most important factors associated with breastfeeding. SSC, when the naked baby is held against the mother’s chest between her breasts, is considered one of the most important factors for successful initiation and continuation of breastfeeding [9]. Using the appropriate posture during breastfeeding is also a significant factor in continuing breastfeeding, particularly in the first 6 months [18].

On the other hand, medication usage, physical and interpersonal tension are identified as inhibiting factors for breastfeeding [19]. Furthermore, remaining in a constant physical position is associated with chronic musculoskeletal disorders (MSDs), including back, knee, and neck disorders, which in turn seem to negatively affect breastfeeding [14]. Improving breastfeeding mothers’ health status is crucially linked to their careful attention to ergonomic breastfeeding.

Interventional ergonomic studies have been conducted primarily on industrial workers and employees and depicted that ergonomic intervention leads to improvement in the working conditions of the participants [20–25]. However, the positive influence of ergonomic training programs and interventions on effective breastfeeding has not been adequately examined up to now, and despite extensive investigations to discover analogous studies, no study with similar results was found. The aims of the present study are first, to assess the influence of ergonomic breastfeeding training on infant growth and second, to assess its influence on MSDs in mothers.

## Materials and methods

### Study design

**Fig. 1:**
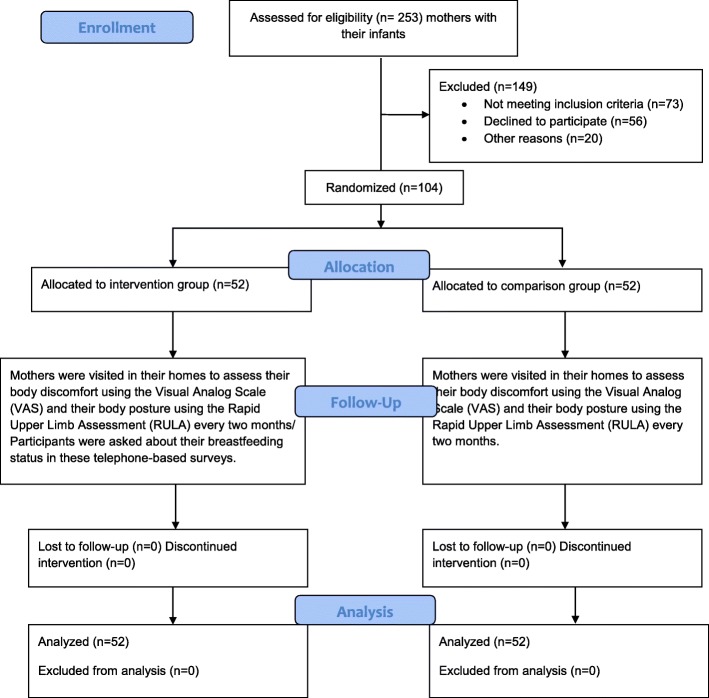
The Fig. 1 doesn’t need legend. The Fig. 1 is the Consolidated Standard of Reporting Trials (CONSORT) flow diagram was described the outline of the design

This randomized controlled trial was conducted in five health centers in Shiraz, capital of Fars province, South-East Iran. All breastfeeding mothers referring to these centers (*n* = 253) were invited to participate after their eligibility for entering the study was assessed. A total of 104 individuals volunteered to take part in the study. To determine the effectiveness of the intervention, the participants were randomly divided into two groups, intervention and comparison groups. The distribution of the infant’s sex was balanced across groups, using group matching. The Consolidated Standard of Reporting Trials (CONSORT) flow diagram was used for the outline of the design (Fig. 1).

All participants partook in the study voluntarily after receiving verbal information about the aims and protocol of the study and confidentiality protocols. The study protocol was approved by the ethics committee of Shiraz University of Medical Sciences (Ethics committee reference number: CT-9379-7439). Additionally, the study was conducted in accordance with the Helsinki Declaration of 2013 [26]. It should be noted that this study was registered in the Iranian registry of clinical trials with a trial registration number of IRCT2014042317398N1.

The participants in the study were trained to take four breastfeeding positions, namely, cross-cradle, under-arm, side-lying, and supine. Each participant chose one of the breastfeeding methods and fed her infant in the selected position. It should be noted that none of the participants chose the supine position.

For all participants, anthropometric measurements were preformed every two months, up to 6 months after childbirth. Participant in the intervention group also participated in face-to-face ergonomic training sections. The training program was provided to each participant individually over 3 sessions each for approximately 15–20 min. In addition, the intervention group were reminded of the breastfeeding position and ergonomics training program for 10 min via regular weekly calls. Participants were asked about their breastfeeding status in these telephone-based surveys.

The ergonomics training included the most ergonomically appropriate methods to breastfeed and ergonomic guidelines, including how to cuddle and breastfeed the newborn correctly. The content of the ergonomic training program was designed by the ergonomist authors. The study protocol was reviewed and approved by faculty members of the ergonomics department of Medical Sciences in Shiraz University.

It is worth mentioning that ergonomic training was provided to the intervention group after they were informed that they would be observed and the ergonomics of their posture would be assessed.

Face-to-face ergonomic training (with regular reminders) and measurements of the infants’ weights and heights were performed at the health centers. Participants were visited in their homes to assess their body discomfort using the Visual Analog Scale (VAS) and their body posture using the Rapid Upper Limb Assessment (RULA).

The content of the ergonomics training package was as follows:

#### Sitting position

In this position, the participant was advised to sit upright and use supports, such as a pillow, for the arm, back, and foot regions. In this situation, the participant would not have to feed the infant while leaning forward or backward (flexion or extension of the trunk). If the participant bends forward, this posture exerts pressure on the back and neck muscles. If her trunk leans back, the breast is farther from the infant and it is difficult for the infant to continue feeding. In addition, it is best if the participant’s chair height is slightly lower than her popliteal height. In this way, the mother’s feet are placed on the floor and her knees are slightly above her thighs. In this position, the infant’s weight is balanced on the trunk, thighs and knees of the mother, and the infant is not hanging on the mother.

#### Cradle position

In this position, it is recommended that the mother puts pillows behind her back (shoulders and lower back), and under the elbow so that she can keep the child high enough. In this situation, light pressure is exerted on the mother’s neck, shoulders, arm, and back muscles.

#### Cross-cradle position

In this posture, it is recommended that 1) the mother sits upright and puts pillows behind her back. In this way, she does not have to bend over the infant. 2) The mother holds the infant horizontal or semi-horizontal on the pillow or cushion on her knees and thighs. 3) If the infant is feeding at the left breast, the mother holds the infant with the right hand and vice versa.

#### Lying position (lying on the side)

In this situation, it is recommended that the mother puts several pillows under her head, back and between her knees to make feeding easier.

### Sample size

The sample size was calculated based on the results of a pilot study. To achieve both study aims, the variable with bigger variance (body discomfort) was selected to calculate the required sample size. Sample size was calculated to detect a 1.2 point difference between the two groups (intervention and comparison) in body discomfort measured by VAS, with an alpha value of 0.05 and a power of 80%. It was determined that the number of participants should be 44 in each group. However, considering a 15% drop out rate, the final sample was increased to 52 in each group.

Accordingly, a list of mothers feeding their neonates was prepared and randomly numbered based on the randomized table. Finally, all 104 participants (with no drop outs) were randomly allocated to the intervention and comparison groups via the size-four block sampling method. The study was conducted according to a single blind protocol. In the way that, both groups of participants were not informed about the assigned group.

#### Inclusion criteria

All interviewed participants had to be breastfeeding, in good general physical health (no discomfort in body regions), and have a normal Body Mass Index (BMI) of 18.5 to 24.9, a healthy newborn, no history of breast cancer or breast surgery, no use of medication affecting breastfeeding, and at least a moderate level of education.

#### Exclusion criteria

This study excluded those with any history of diseases affecting milk volume or the child’s weight and height and mothers not willing to continue EBF for the first 6 months of the child’s life.

### Measures

To meet aims of the study used following measures:

#### Assessment of the impact of ergonomic breastfeeding training on infant growth

##### Demographic questionnaire

The first part of the questionnaire included questions about the mother’s demographic and anthropometric characteristics such as age, weight, height, sex, education (high school diploma and university graduate), number of children, income, type of delivery, birth order, and breastfeeding method. The infant’s anthropometric features, such as weight and height, were included in the second part of the demographic questionnaire.

#### Influence of ergonomic breastfeeding training on MSDs in participants

##### Body map

The site of discomfort was determined on a body map. The map divided the body into 9 regions. The participants were asked to mark the site of their discomfort on the body map [27].

##### Visual analog scale

This tool was used to assess the severity of discomfort in the participants’ body regions. The VAS is a single-item measure that consists of a 100 mm horizontal line anchored by two opposite labels (0 = no discomfort and 100 = maximum discomfort). Participants marked their discomfort score on the scale using a slash. This gave them the greatest opportunity to choose the accurate severity of their discomfort [28, 29]. The validity and reliability of the VAS for the assessment of chronic musculoskeletal pain has been surveyed by Boonstra et al. using a test-retest design (r = 0.60–0.77 for all body regions) [30].

##### Rapid upper limb assessment

The RULA was used to assess the mothers’ body posture. The RULA was developed by McAtamney and Corlett to assess the upper extremity MSDs risk level. In this technique, the postural and biomechanical load at different body regions, such as the upper extremities, neck, trunk, and legs, is considered [31].

In the RULA technique, the body segments are divided into two sections: “A” (arms, forearms, and wrists) and “B” (neck, trunk, and legs). In this technique, it is specified that static and awkward postures of the “B” section might impact the assessment of the body regions of the “A” section.

To assess the body postures using the RULA, first the score for group “A” and second the score for group “B” were determined for both the right and left parts of the body. A graph based on the posture scoring scale, with some additional adjustments, was used to calculate each group score (A and B). Additionally, the posture of the arm, forearm, and wrist were assessed by observation and their values were calculated by inserting the measured values in the diagram. Then, the status of the neck, trunk, and lower limbs were also assessed by the diagram and their relevant values were determined. The mutual impact of these limb postures determined the risk of incidence of MSDs. To determine the influence of force, muscular activity, and repetition of the movement, the relevant tables were used and the final score, ranging from 1 to 7, was determined by combining the values. The greater the score, the higher the risk of MSDs. This score was calculated for each side of the body separately. The action level, determined through the RULA method, was classified into the following four categories: Action level 1: low level of risk (change may be needed), Action level 2: moderate level of risk (change is needed), Action level 3: high level of risk (immediate action), and Action level 4: very high level of risk (full immediate action). Previous studies have indicated that there are significant relationships between individual RULA body part scores and the development of pain or discomfort in those body regions. Additionally, the reliability test of the RULA technique determined that there was a high scoring consistency among different individuals. This technique has been used repeatedly to assess the risk for MSDs in various ergonomics studies [32, 33].

### Data collection

Data collection was carried out from March to September, 2017. Information about the mothers, who were clients of some health centers, and their infants was obtained through both their self-record and a questionnaire. The aims of the study were explained to the participants, and formal consent was obtained before the interview. An interview-administered questionnaire was used to collect data for each participant. The interview was conducted by a trained ergonomist who measured the anthropometric indices (weight and height) of the newborns at the end of the interview. Infants were weighed with minimal clothing and without shoes using a calibrated LAVITA Platform Balance Scale, and the numbers were rounded to the nearest 10 g. In infants, height was measured from the top of the head to the heel of the foot using a length board while the infants were in a recumbent position. The height figures were rounded to the nearest millimeter. As participants self-reported, they breastfed their neonates on average 12 to 15 times every day for approximately 10 min each time.

During the 6 months of the study, the weight and height of the neonates were measured every 2 months and growth charts were drawn.

In the intervention group, EBF participants were examined by a trained ergonomist to improve their position and maintain the optimal posture regarding their neck, trunk, legs, arms and wrists (for the left and right sides) during the breastfeeding process. Ergonomic training was conducted based on the participants’ different breastfeeding methods. For instance, in the under-arm position, it was suggested to the mothers that they put their arm above the baby’s head, bend it under their own head and cover the infant with the other hand to pull the baby close. Subsequently, in the intervention group, mothers were asked if they had difficulty with recommended ergonomic postures and by ergonomists the problems were fixed. The protocol for the training was prepared and assessed by an expert panel consisting of an ergonomist, a physiotherapist and a health education specialist. In addition, verbal ergonomic training and direct observation were provided for the intervention group. For the comparison group, informational booklets were given to the EBF participants, and direct monitoring was performed. The RULA and VAS were administered in mothers at 2, 4 and 6 months after childbirth, and the intervention was started within 3–5 days after childbirth and continued up to 6 months. In the RULA technique, the posture of the arm, forearm, and wrist breastfeeding mothers were assessed by observation and their values were calculated. Final score was calculated by ergonomists and action level was determine. Mothers marked their discomfort score on the VAS scale to assess the severity of discomfort in their body regions.

### Data analysis

Data were analyzed by Statistical Package for Social Sciences 19 (SPSS Inc., Chicago, IL, USA) using Independent sample t-test and Chi-squared test. *P*-value < 0.05 was considered statistically significant [34]. To adjust the significant level (*α*), as the number of tests increase, the Bonferroni correction was use [35].

#### Assessment of the impact of ergonomic breastfeeding training on infant growth

Independent sample t-test was used to compare intervention and comparison groups for infants’ heights and weights at the baseline (birth) and the mean difference of the above indexes at birth and 6 months of age. Paired t-test was used to examine the mean difference in the infants’ height and weight between birth and 6 months after for the intervention and comparison groups, separately.

#### Influence of ergonomic breastfeeding training on MSDs in participants

Chi-squared test was used to examine the association of qualitative characteristics and MSD risk level derived from the RULA technique between both groups. Independent sample t-test was used to compare the discomfort at each time point based on the Visual Analog Scale (mean, SD) between intervention and comparison groups.

## Results

The demographic characteristics of the participants are presented in Table 1.

**Table 1:** Baseline Characteristics of the individuals in the intervention and comparison groups participating in the study on influence of ergonomic breastfeeding training on some health parameters in infants and mothers (*n* = 52)

Qualitative variable	Intervention	Comparison	*P*-value
Mothers’ Education n (%)			
High School Diploma	38 (73.08)	29 (55.77)	0.06*
University Graduate	14 (26.92)	23 (44.23)	
Mothers’ Job n (%)			
Employed	14 (26.92)	16 (30.77)	0.66*
Housewife	38 (73.10)	36 (69.20)	
Mothers’ Age			
**Mean (SD)**	29.40 (5.80)	27.60 (5.00)	0.67**
Mothers’ BMI in the first month of pregnancy			
**Mean (SD)**	23.12 (0.90)	23.34 (1.97)	0.87**
Type of delivery n (%)			
Vaginal delivery	46 (88.46)	48 (92.31)	0.50*
Cesarean section	6 (11.54)	4 (7.69)	
Birth order n (%)			
First	27 (51.92)	33 (63.46)	0.23*
Second and higher	25 (48.08)	19 (36.54)	
Neonate^’^s gender n (%)			
Girl	31 (59.62)	31 (59.62)	–
Boy	21 (40.38)	21 (40.38)	

* Chi-square test at α = 0.0125 (Bonferroni correction)

** Independent samples t-test at α = 0.025 (Bonferroni correction)

As shown in this table, confounding variables compared between intervention and comparison groups and none of the variables is not significant (*p* > 0.05).

### Assessment of the impact of ergonomic breastfeeding training on infant growth

Table 2 shows the comparisons of infants’ weight-for-age (kg), height-for-age (cm) and weight-for-height between the intervention and comparison groups (*N* = 104) based on WHO child growth standards Z score [36]. As shown in the table (Table 2) the difference of weight-for-age and weight-for-height of infants (both girl and boy) at different waves of measurements (at birth, 2, 4, and 6 months post birth) were not statistically significant between intervention and comparison groups. The only statistically significant result was for height-for-age, in the 6 months post birth among the girls (*p* < 0.01).

**Table 2:** A Comparison of Infants^’^ Weight-for-Age (kg), Height-for-Age (cm) and Weight-for-Height between the intervention and comparison groups participating in the study on influence of ergonomic breastfeeding training on some health parameters in infants and mothers based on World Health Organization Child Growth Standards Z Score (Girl = 62, Boy = 42)

Variable			Intervention (*n* = 52) M (SD)	Comparison (*n* = 52) M (SD)	*P*-value
Weight-for-age	At birth	Girl	0.10(1.04)	0.20(0.92)	0.69
		Boy	−0.12(0.91)	0.11(1.00)	0.43
	2 months post birth	Girl	0.13(0.65)	0.27(0.61)	0.37
		Boy	−0.55(0.65)	− 0.44(0.62)	0.59
	4 months post birth	Girl	1.02(0.56)	1.02(0.70)	0.96
		Boy	0.36(0.54)	0.33(0.61)	0.89
	6 months post birth	Girl	2.09(0.91)	2.40(0.49)	0.10
		Boy	1.75(0.56)	1.44(0.94)	0.21
Height-for-age	At birth	Girl	0.34(0.88)	0.41(0.82)	0.75
		Boy	−0.50(1.11)	−0.11(0.71)	0.19
	2 months post birth	Girl	0.80(0.90)	0.57(0.85)	0.32
		Boy	−0.21(1.04)	−0.52(1.37)	0.41
	4 months post birth	Girl	0.90(1.08)	0.43(1.37)	0.13
		Boy	−0.33(1.32)	−0.20(1.25)	0.73
	6 months post birth	Girl	1.68(1.20)	0.78(1.53)	0.01
		Boy	0.29(1.27)	0.38(1.52)	0.84
Weight-for-height	At birth	Girl	−0.08(1.05)	0.01(0.93)	0.69
		Boy	−0.40(1.25)	0.02(0.80)	0.20
	2 months post birth	Girl	−0.08(1.03)	0.14(0.96)	0.37
		Boy	−0.07(1.00)	−0.36(1.31)	0.41
	4 months post birth	Girl	−0.05(0.93)	−0.04(1.15)	0.96
		Boy	−0.09(1.02)	0.012(0.98)	0.73
	6 months post birth	Girl	−0.21(1.22)	0.20(0.65)	0.10
		Boy	−0.06(0.86)	0.00(1.03)	0.84

Independent samples t-test at α = 0.002 (Bonferroni correction)

### Influence of ergonomic breastfeeding training on MSDs in participants

No significant difference between the priority level for corrective action was found between the groups before the intervention (*p* > 0.05). However, a significant association between training and the priority level for corrective action was found at 2, 4 and 6 months post childbirth (*p* < 0.001) (Table 3). The table displays the difference between the two groups in the RULA categories and indicates that the participants in the intervention group were at lower risk of MSDS. The results of the body map were compared and the results are presented in Table 4. Compared to the comparison group, the intervention group reported less back pain (13.49% vs. 30.76%, *p* = 0.03) 6 months post childbirth. Indeed, the number of mothers with back pain was reduced among the intervention group 4 months after infants birth, though this difference was not statistically significant. In addition, compared with the intervention group, the number of participants with both back and neck pain was larger in the comparison group at 2, 4 and 6 months post childbirth (*p* > 0.05 for all). As shown in this study, the mean discomfort score in the intervention group was lower at 4 months post the birth (1 ± 0.90) rather than comparison group (1.70 ± 1.50) and 6 months post childbirth in the intervention group (0.90 ± 1.05) rather than comparison group (1.85 ± 1.60) (*p* < 0.05). Findings indicated that the comparison group experienced higher levels of discomfort at 4 and 6 months (Table 5).

**Table 3:** Comparison of the priority level of corrective action determined using the RULA method for mothers in both the intervention and comparison groups participating in the study on influence of ergonomic breastfeeding training on some health parameters in infants and mothers (n = 104)

Variable	Action level*	Intervention (*n* = 52) n (%)	Comparison (*n* = 52) n (%)	*P*-value
At birth	1	8 (15.38)	14 (26.92)	0.14
	2	20 (38.46)	25 (48.08)	
	3	20 (38.46)	11 (21.15)	
	4	4 (7.69)	2 (3.85)	
2 months post birth	1	39 (75.00)	15 (28.85)	0.001
	2	10 (19.23)	30 (57.69)	
	3	3 (5.77)	7 (13.46)	
	4	0 (0.00)	0 (0.00)	
4 months post birth	1	43 (82.69)	18 (34.62)	0.001
	2	6 (11.54)	25 (48.08)	
	3	3 (5.77)	9 (17.31)	
	4	0 (0.00)	0 (0.00)	
6 months post birth	1	43 (82.69)	16 (30.77)	0.001
	2	7 (13.46)	28 (53.85)	
	3	2 (3.85)	8 (15.38)	
	4	0 (0.00)	0 (0.00)	

Chi-square test at α = 0.0125 (Bonferroni correction)

*Action level 1: low level of risk (change may be needed), Action level 2: moderate level of risk (change is needed), Action level 3: high level of risk (immediate action), and Action level 4: very high level of risk (full immediate action)

**Table 4:** The Site of Discomfort Reported by the Two Groups Based on the Body Map (*N* = 104)

Variable	Intervention (*n* = 52) n (%)	Comparison (*n* = 52) n (%)	*P*-value
2 months later childbirth			
Neck	11 (21.15)	10 (19.23)	0.80
Back	9 (17.30)	13 (25)	0.33
Knee	11 (21.15)	8 (15.40)	0.44
4 months later childbirth			
Neck	13 (25)	13 (25)	1.00
Back	7 (13.46)	15 (28.85)	0.05
Knee	12 (23.07)	11 (21.15)	0.81
6 months later childbirth			
Neck	13 (25)	11 (21.15)	0.64
Back	7 (13.46)	16 (30.76)	0.03
Knee	8 (15.38)	12 (23.07)	0.32

Chi-square test at α = 0.006 (Bonferroni correction)

**Table 5:** Comparison of Discomfort at Each Time Point Based on the Visual Analog Scale* (*N* = 104)

Variable	Interventional (*n* = 52) M (SD)	Comparison (*n* = 52) M (SD)	*P*-value
2 months post birth	0.87 (1.05)	1.40 (1.55)	0.17
4 months post birth	1 (0.90)	1.70 (1.50)	0.02
6 months post birth	0.90 (1.05)	1.85 (1.60)	0.001

Independent samples t-test at α = 0.017 (Bonferroni Correction)

*The possible range of the VAS is 0–100 (0 = no discomfort and 100 = maximum discomfort)

## Discussion

Interpretation of the study findings are as follows:

### Assessment of the impact of ergonomic breastfeeding training on infant growth

This study with only one exception suggested non-significant differences of the infants’ height and weight between the intervention and comparison groups. In general, the results of the current study indicated that the ergonomic breastfeeding intervention had no effect on the two most important anthropometric indices (height and weight) of the infants. In this context, mothers were advised to continue breastfeeding until the baby was fully nourished, otherwise ethical issues in the study were not respected. This issue can be related to the fact that ergonomics training to the mothers has not been very important issue in growth (increasing of the weight and height) of the infants. Based on our research, influence of ergonomic interventions on effective breastfeeding has not been adequately examined up to now; although, in the previous studies have revealed that the volume of the milk, time of the breastfeeding are significant factors for the growth of the infants [18, 23].

### Influence of ergonomic breastfeeding training on MSDs in participants

The current investigation was an interventional study that examined the association between anthropometric factors and the effectiveness of a training program.

A key finding of the present study was the positive impact of training on participants’ upper limb pain; based on the results, training would improve the health of most participants. For instance, in the intervention group, the number of mothers who were at moderate and high risk levels of MSDs decreased with time (after 2, 4 and 6 months after childbirth), while in the comparison group, an increasing trend was observed. This finding is in agreement with Stock et al.. Stock and colleagues showed that ergonomic training could play an important role in improving mothers’ knowledge, which would then affect their behavior and improve their sitting posture in the workplace [37].

According to the VAS results, the intervention group reported lower scores for MSD and pain, which is similar to a study by Myles et al. [38] showing that ergonomic training was effective in reducing MSDs, including back, knee and wrist pain. Likewise et al. [39] found an inverse association between ergonomic training and low back and lumbar pain among the participants who were in the intervention group who used a back support. Additionally, in our study, the participants who received the intervention felt less discomfort, especially 4 and 6 months post childbirth.

The results of the current study, presented in Tables 3 and 4, suggest that the training program provided to participants lowers the likelihood of ergonomic disorders such as the risk of MSDs and the severity of pain/discomfort in the future. This finding agrees with previously published studies [40]. According to the RULA results, the number of participants who reported suffering from backache was reduced over time after the intervention. Compared to the comparison group, participants in the intervention group reported less knee pain after 6 months. The results of the present study show that VAS scores for both back and knee pain were reduced in the intervention group, from 9 to 7 and 11 to 8, respectively. Finally, our results concur with Mahmud, Kenny, MdZein, and Hassan’s findings [41], which found that proper ergonomics training could have a positive impact on humans, to reduce MSDs by changing unfavorable posture habits.

In this blind study, random assignment of participants did not encourage individuals to be in a particular group and therefore, the distribution of confounding variables such as external intervention (midwives, pediatricians, peer-support group …) in both groups is identical.

### Limitations of the study

Following participants in the process of feeding their neonates was difficult because they did not visit the health centers regularly. Although the results of this study indicate that ergonomic training during breastfeeding can lead to effective breastfeeding, the randomization protocol was unsuccessful in properly randomizing education levels, so the findings should be interpreted cautiously. Due to the small sample size, cultural and racial diversity may change the results of the present study, so it is not generalizable to all mothers. Only mothers who exclusively breastfed participated in the study, so there was no information about the quantity of formula use. The results of this study were adjusted for possible confounders (e.g. maternal education),which are known to influence child’s growth. Although the results suggested that ergonomic training did not improve the mother’s satisfaction about BF, future investigations are needed to explain the reasons behind the results.

In this study, the lying down breastfeeding position was not selected by any participants. This issue should be addressed in future studies.

## Conclusion

This study provides evidence for effectiveness of ergonomic positions of breastfeeding in mother’s musculoskeletal disorders and discomfort. Exclusive breastfeeding is one of the most important health issues worldwide; therefore, it deserves to receive more attention with regard to the mother’s and child’s health.

## Data Availability

Not applicable.

## Abbreviations

BMI: Body Mass Index
CONSOR: Consolidated Standard of Reporting Trials
EBF: Exclusive breastfeeding
MSDs: Musculoskeletal disorders
RULA: Rapid Upper Limb Assessment
SSC: Skin-to-skin contact
VAS: Visual Analog Scale
